# Hal-Py-SO_3_H as a novel and recyclable catalyst for highly efficient synthesis of xanthene and spiropyran derivatives

**DOI:** 10.1038/s41598-024-58647-x

**Published:** 2024-04-06

**Authors:** Mohammad Abdolmaleki, Mansoureh Daraie, Zohreh Mirjafary

**Affiliations:** Department of Chemistry, Science and Research Branch, Islamic Azad University, Tehran, Iran

**Keywords:** Halloysite, Nano catalyst, Spiropyrans, Acidic catalyst, Xanthene, Catalyst synthesis, Heterogeneous catalysis, Organocatalysis

## Abstract

The aim of this research is to synthesize a new sulfonic acid catalyst based on halloysite nanotubes (Hal-Py-SO_3_H) and characterize it as a solid acid nanocatalyst by various analytical techniques such as Fourier-Transformed Infrared spectroscopy (FTIR), Thermal gravimetric Analysis (TGA), X-ray Diffraction (XRD) analysis, Scanning Electron Microscopy (SEM), Vibrating Energy-Dispersive X-ray analysis (EDX), Transmission electron microscopy (TEM) and X-ray atomic mapping. Furthermore, this new catalyst was evaluated in synthesizing spiropyran derivatives via multicomponent reactions (MCRs) and Xanthen derivatives under environmentally sustainable conditions. The main advantages of this approach include green conditions, excellent yields, quick reaction rates, and ease of preparation. Additionally it was observed that the catalyst exhibited robust stability even after multiple recycling processes, indicating its potential for practical applications in sustainable chemical transformations.

## Introduction

Green chemistry principles (GCP) were introduced by Paul Anastas and John Warner with the intention of encouraging the adoption of eco-friendly chemical processes and products. The primary objective of GCP is to mitigate pollution through the reduction of hazardous chemical usage and waste generation. These principles have found extensive application across various sectors including industry, governmental regulations, educational institutions, and advancements in technology. GCP emphasizes the development of safer chemicals, the use of catalysts instead of stoichiometric reagents, and waste prevention^1^.

Catalysts play a crucial role in the majority of processes within the contemporary chemical industry due to their ability to mitigate the risks involved in the preparation and utilization of diverse chemicals^2^ .Catalysts are of utmost importance in various chemical protocols conducted in both academic and industrial research laboratories^3^. These protocols aim to enhance efficiency, yield, and selectivity in numerous chemical processes^4^. The presence of catalysts is indispensable in the manufacturing process of numerous items, encompassing pharmaceuticals, fine chemicals, polymers, fibers, fuels, paints, lubricants, and an extensive assortment of value-added products^3^. The use of eco-friendly materials, such as solid acids, can facilitate the advancement of cleaner technologies. Solid acid catalysts provide several benefits over liquid acid catalysts. They are non-corrosive and environmentally friendly, making disposal easier. Therefore, the utilization and investigation of solid and green catalysts are crucial in promoting organic syntheses^5^.

Hydrated aluminosilicates known as clay nanotubes (HNTs) belong to the kaolinite group^6^ and exhibit unique meso/macroscopic superstructures. These structures are characterized by hollow cylinders formed by the layering of octahedral gibbsite Al(OH)_3_ and tetrahedral SiO_4_ sheets, resulting in the formation of halloysite nanotubes^7^. The wrapping of these sheets around each other occurs under specific geological conditions, giving rise to the distinctive halloysite structure^8,9^.

Clay nanotubes (HNTs) have attracted significant interest in contemporary scientific investigations due to their distinctive characteristics, which encompass elevated specific surface areas and the ability to undergo functionalization. These nanotubes are utilized in a range of fields, such as drug delivery, nanoreactors^10^, and ion exchange membranes^11^. Diverse techniques, including nanoparticle depositions and surface alterations, have been utilized to enhance and tailor the properties of HNTs^6^.

Spiroheterocyclic compounds synthesized from ninhydrin through multicomponent reactions have become increasingly important in the field of medicinal chemistry because of their varied biological properties. In recent times, there has been a significant focus on spiro compounds owing to their extensive range of biological activities^12^. In several studies, spiropyrans have been found to possess anti-leishmanial properties, hypotensive properties, analgesic properties, antitumor properties^12^, Anticonvulsant^13^, antioxidant, antimicrobial and antibacterial^14^, anti-HIV^12,13,15^, anticonvulsant^16^ and anticancer^13–16^ properties. In addition, spiropyrans are effective in treating Alzheimer's disease and schizophrenia^12^.

**Figure 1:**
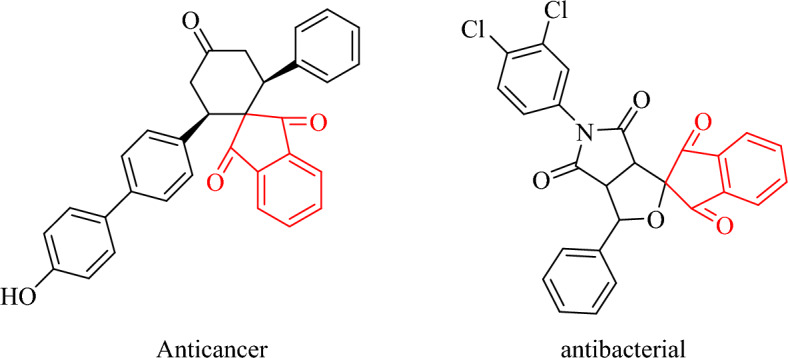
Representative examples of bioactive compounds.

Multicomponent reactions (MCRs) have become increasingly popular among chemists due to their ability to synthesize intricate molecules using easily accessible raw materials^17^. These reactions possess several advantages over sequential synthesis methods, including minimal waste production and the absence of unwanted by-products. MCRs offer step economy, high convergence, and structural diversity, making them an environmentally friendly approach for the synthesis of biologically active compounds in the pharmaceutical industry. While MCRs have long been acknowledged for their ability to generate diverse organic structures, they have recently garnered increased attention^18^.Within MCRs, ninhydrin, a tricarbonyl compound, has emerged as a valuable tool, contributing to the development of diverse structural scaffolds with heterocyclic properties. It can be seen two examples of compounds with differing biological activities in Fig. 1^19^.

Xanthenes are essential heterocyclic compounds found in both natural products and pharmaceuticals, serving a significant function^20^. These compounds demonstrate a diverse array of effects, such as antiviral, antibacterial^21^, antimicrobial, antiproliferative, and antioxidant properties. Additionally, derivatives of xanthene hold great significance in the development of laser dyes, fluorescent sensors, and protein labeling, playing a crucial role in laser technology and the observation of biomolecular processes^20^.

Synthetic organic chemistry plays an important role in the advancement of environmentally friendly chemical reactions, which are essential for the sustainable production of various chemicals. With the increasing concerns regarding environmental and safety issues, the demand for innovative technologies has grown significantly. Heterogeneous catalysts have emerged as a promising solution due to their ability to minimize waste generation, decrease the reliance on hazardous materials, and facilitate easy separation and reusability. As a result, there is a substantial demand for these catalysts across various industries and applications^22,23^.

In comparison to heterogeneous catalysts, homogeneous catalysts possess superior catalytic activity and selectivity. However, the separation of homogeneous catalysts from reaction mixtures presents a significant challenge. In organic and green chemistry, the emphasis is on the development of highly active and selective heterogeneous catalysts. This is due to the fact that surface-dependent catalysis relies on a substantial surface area and numerous active sites. Catalysts with excellent dispersion and high surface areas prove to be beneficial in this context. Therefore, the development of an effective method to increase the quantity of active sites and surface area in nanoparticles is necessary^23,24^.

**Figure 2:**
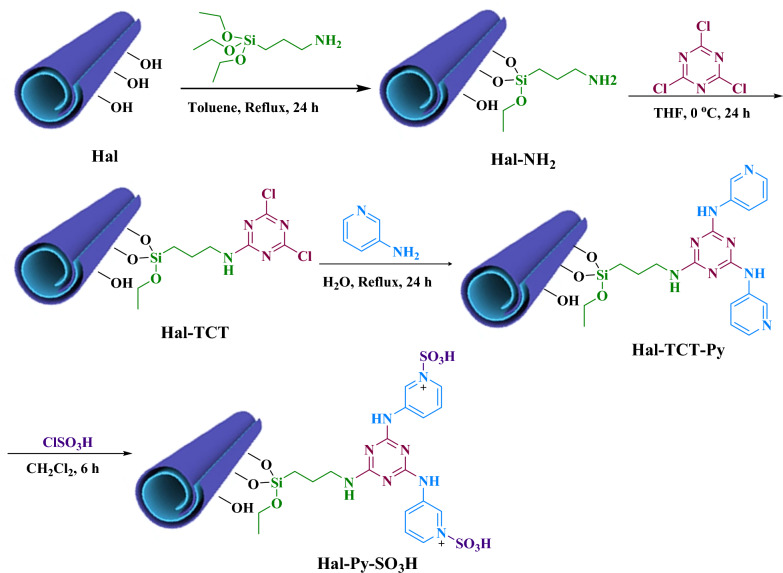
The synthesis pathway of Hal-Py-SO_3_H.

There has been an increasing emphasis on adopting more environmentally sustainable approaches for the synthesis of chemicals due to growing environmental apprehensions. Conversely, eco-friendly catalysts have been engineered without the use of metals^25^. The present study examines the synthesis and application of a new catalyst in a well-known reaction to evaluate its efficacy in comparison to other catalysts. This specific catalyst demonstrates higher acidity levels due to the existence of two active sites of TCT. One notable development involves the covalent conjugation of high concentrations of sulfonic acid with Halloysite nanoclay, producing new Brønsted acid catalysts. This environmentally friendly approach is illustrated in Fig. 2.

## Experimental

### Materials and instruments

For the preparation of the catalyst, reagents and solvents were used as follows: Halloysite (Hal), 3-(aminopropyl)-triethoxysilane (APTES), 2,4,6-trichloro-1,3,5-triazine (TCT), 3-Aminopyridine, K_2_CO_3_, toluene, THF, CH_2_Cl_2_, Chloro sulfunic acid, were purchased from Sigma-Aldrich.

A variety of substances have been evaluated for their catalytic capability and efficiency, including: malononitrile, Ethyl cyanoacetate, benzaldehyde derivatives, various of 1,3-dicarbonyl compounds such as dimedone, 4-hydroxy coumarin, 4-hydroxy-6-methyl-2-pyrone and etc., were procured from Sigma–Aldrich.

Several analyses were conducted to confirm the structure of the catalyst, including: X-ray diffraction (XRD), Fourier transform infrared (FTIR), scanning electron microscope (SEM), Transmission electron microscopy (TEM), thermos gravimetric analysis (TGA), energy dispersive spectroscopy (EDS) and elemental mapping analyses.

### Synthesis of Hal-Py-SO_3_H

The synthesis of Hal-Py-SO_3_H nano catalyst involves several steps as follows (Fig. 2):

- Hal Functionalization with APTES (Hal-NH_2_): Hal (2 g) was dispersed in dry toluene (25 mL) for 20 min. After adding the APTES to the Hal suspension, the mixture was refluxed under N_2_ atmosphere for 24 h. Then the mixture was filtered and the obtained solid was washed with dry toluene and dried at 60 °C for 8 h^26^.
- Preparation of Hal-TCT: Hal-NH_2_ (1.5 g) was dispersed in dry THF (15 mL), and ultrasonic waves were applied for 20 min. After homogenizing the mixture, it was stirred for 30 min in an ice bath. After adding TCT (1.2 g in 10 mL dry THF), the mixture was stirred for a day at 0 °C. A conventional filter was used to collect the precipitate, which was washed with THF and dried for 8 h at 60 °C^26^.
- Synthesis of Hal-Py: Hal-TCT (1.2 g) in deionized water (10 mL) was well dispersed under ultrasonic irradiation for 10 min. Then, 3-aminopyridine (1.2 g in 10 mL deionized water) and K_2_CO_3_ (0.5 g) were added to the Hal-TCT suspension, and the mixture was refluxed overnight at 100 °C under inert gas. The solid was filtered, rinsed several times with water, and dried for 8 h at 60 °C.
- Synthesis of Hal-Py-SO_3_H: Hal-Py (1 g) was dispersed in dry CH_2_Cl_2_ (15 mL) for 10 min under ultrasonic irradiation. The homogenized mixture was stirred in an ice bath for 30 min and then chlorosulphonic acid added dropwise and stirred for 6 h. The precipitate was filtered off, rinsed with CH_2_Cl_2_, and dried at 60 °C for 8 h.

### General procedure for the synthesis of xanthene derivatives

40 mg of catalyst and 5 mL of distilled water were poured into a 25 mL flask. 2 mmol of dimedone and 1 mmol of benzaldehyde derivatives were added to the reaction vessel and was refluxed for 30–100 min by a magnetic heater stirrer. The progress of the reaction was monitored by thin layer chromatography (TLC). After the reaction was completed, the precipitate was filtered and dried. Recrystallization in hot ethanol gave the pure product which identified by melting point (Table 2).

### General procedure for the synthesis of spiropyran derivatives

1 mmol of ninhydrin, 1 mmol of malononitrile/ethyl cyanoacetate and 1 mmol of various 1,3-diketone compounds, were mixed in 5 mL of distilled water as a solvent in the presence of 40 mg of catalyst. The mixture was refluxed for 10–80 min by a magnetic heater stirrer. The reaction progress was monitored by TLC. After the completion of the reaction, the precipitate was filtered and dried. Recrystallization in hot ethanol resulted the pure product which identified by melting point (Table 4).

### Spectral data of the selected synthesized compounds

9-(4-chlorophenyl)-3,3,6,6-tetramethyl-3,4,5,6,7,9-hexahydro-1H-xanthene-1,8 (2H) -dione (**3c**), m.p.: 227–232 °C. FT-IR (KBr, cm^−1^): 2952, 2876, 1636, 1364, 1196, 842. ^1^H NMR (400 MHz, Chloroform-*d*) δ 7.25 (d, *J* = 8.2 Hz, 2H), 7.20 (d, *J* = 8.2 Hz, 2H), 4.73 (s, 1H), 2.48 (m, 4H), 2.26 (d, *J* = 16.3 Hz, 2H), 2.19 (d, *J* = 16.3 Hz, 2H), 1.12 (s, 6H), 1.01 (s, 6H).

2-amino-7,7-dimethyl-1ʹ,3ʹ,5-trioxo-1ʹ,3ʹ,5,6,7,8-hexahydrospiro [chromene-4,2ʹ-indene]-3-carbonitrile (**7a**), m.p.: 290–292 °C. FT-IR (KBr, cm^−1^): 3373, 3308, 3345, 3191, 2953, 2880, 2189, 1710, 1661, 1594, 1464, 1218. ^1^H NMR (400 MHz, DMSO-*d*_6_) δ 6.92–6.51 (m, 4H), 5.03 (s, 2H), 2.56 (d, *J* = 16.0 Hz, 1H), 2.34 (d, *J* = 17.2 Hz, 1H), 2.23 (d, *J* = 16.0 Hz, 1H), 2.05 (d, *J* = 16.0 Hz, 1H), 1.05 (s, 3H), 0.98 (s, 3H).

## Result and discussion

### Catalyst formation verification

**Figure 3:**
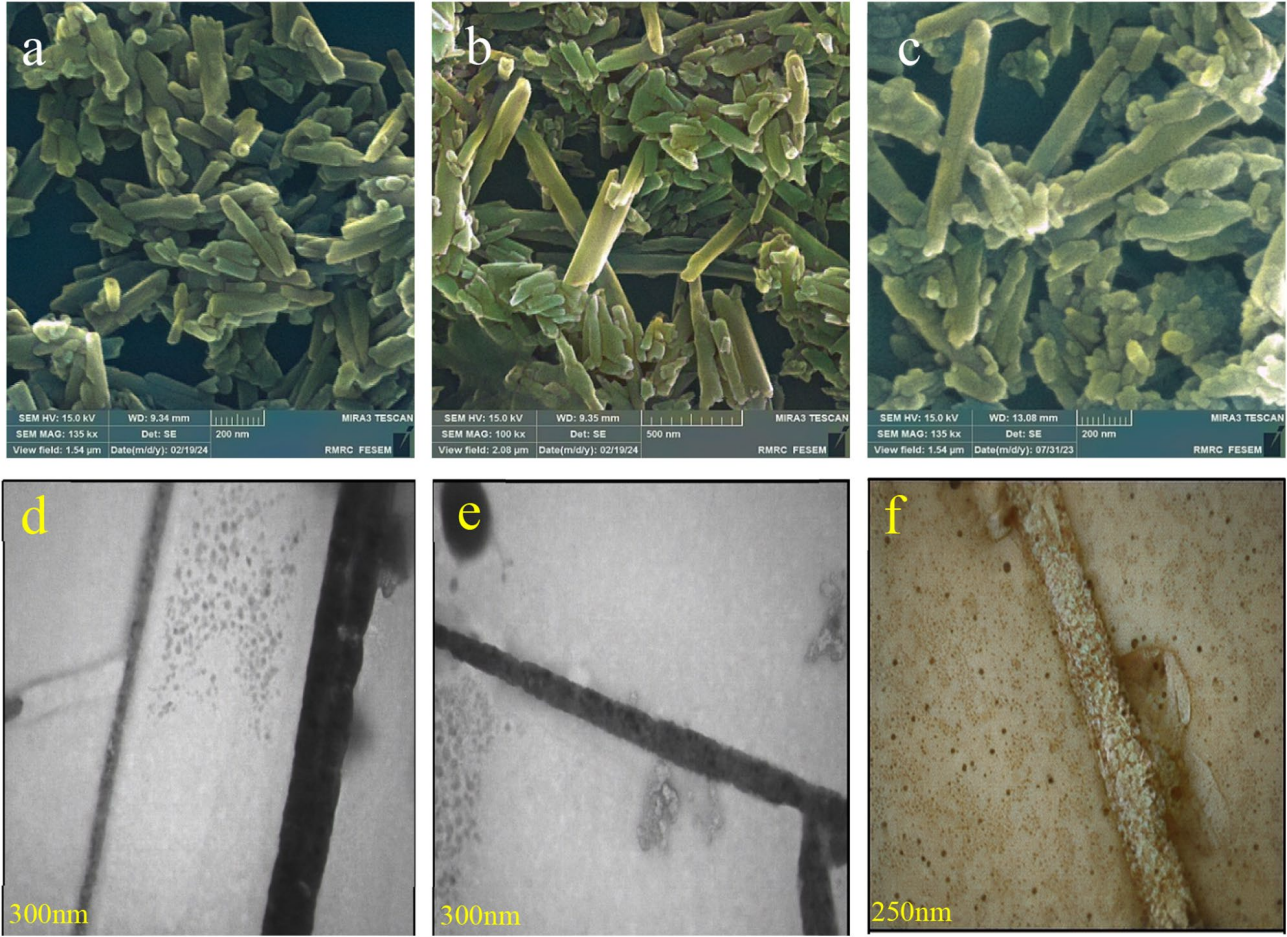
SEM images of (**a**,**b**) Hal, (**c**) Hal-Py-SO_3_H and TEM images of (**d**) Hal, (**e**,**f**) Hal-Py-SO_3_H.

Characterization was conducted on the Hal-Py-SO_3_H as prepared. The morphology of the catalyst was evaluated by SEM image (Fig. 3), The SEM image shows the nanotubes of Hal and confirm that they are not collapsing upon introduction of TCT-Py. Moreover, small aggregates of TCT-Py were observed on the surface of the Hal tubes, indicating their interaction. Figure 3d displays the TEM image of halloysite, which reveals the unique structure of this 1:1 clay mineral resembling nanotubes. Furthermore, the TEM image of the Hal-Py-SO_3_H nanocomposite confirms the existence of halloysite nanotubes.

**Figure 4:**
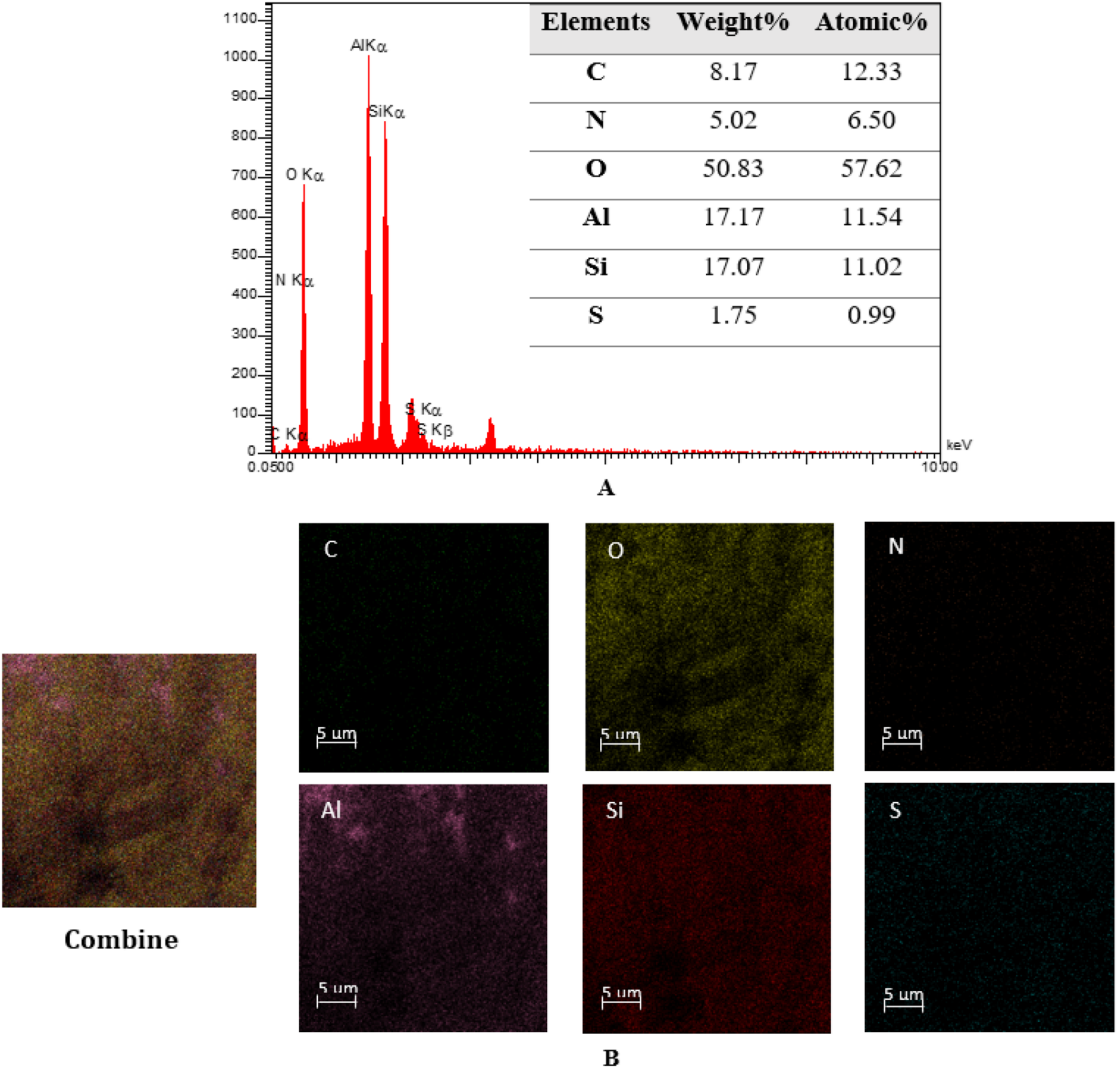
(**A**) EDS and (**B**) elemental mapping analysis of Hal-Py-SO_3_H.

The EDS analysis was conducted to verify the conjugation of Hal-Py-SO_3_H on Hal clay. However, it is important to note that relying solely on a detailed EDS analysis is insufficient to confirm the structure of Hal-Py-SO_3_H, as shown in Fig. 4. The presence of S atoms, as well as C and N atoms, provides evidence for the existence of Py-SO_3_H. Conversely, the absence of Cl atoms in the EDS analysis suggests the successful occurrence of a nucleophilic substitution reaction between TCT and 3-aminopyridine. The elemental mapping in Fig. 4 illustrates that the S, C, and N atoms are evenly distributed, indicating the homogeneous formation of Py-SO_3_H on the Hal clay.

**Figure 5:**
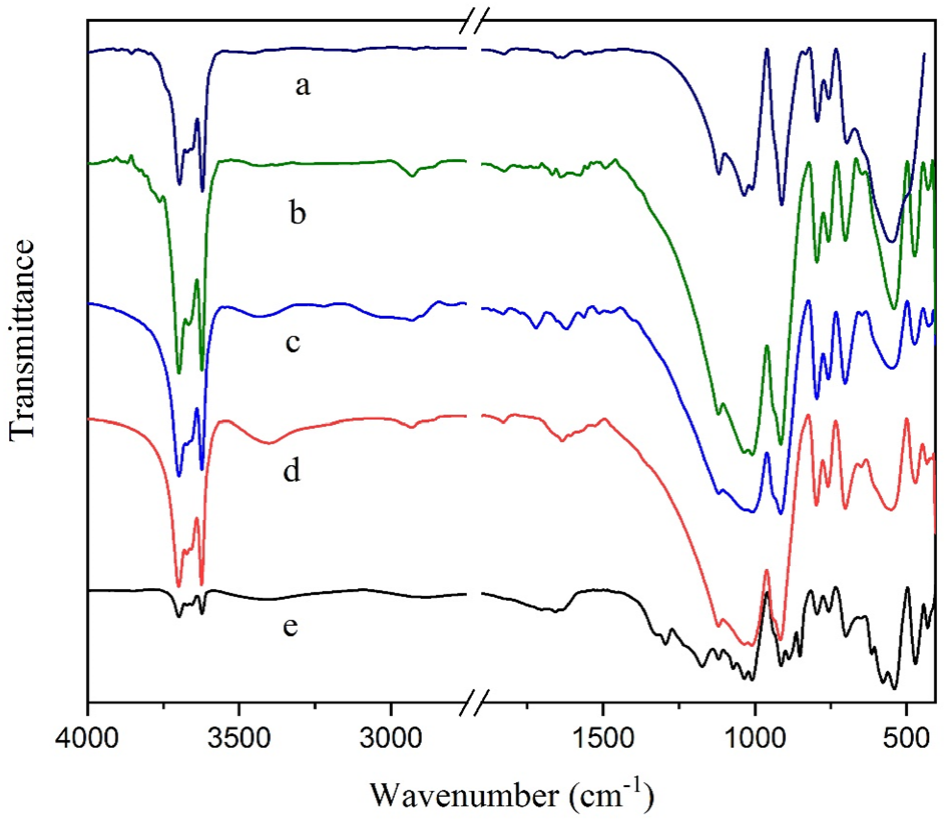
FTIR spectra of Hal (a), Hal-NH_2_ (b), Hal-TCT (c), Hal-TCT-Py (d), Hal-Py-SO_3_H (e).

In Fig. 5, FTIR spectra recorded for Hal, Hal-TCT, Hal-TCT-Py, and Hal-Py-SO_3_H are compared. As reported in the literature^27^, Hal shows absorption peaks at 1055 cm^−1^ (Si–O), 3696 cm^−1^ and 3621 cm^−1^ (inner-OH), and 547 cm^−1^ (Al–O–Si). The FTIR spectra of Hal-TCT, Hal-Py, and Hal-Py-SO_3_H show these characteristic bands, which confirm the stability of Hal structure during the incorporation of organic species. In the FTIR spectra of Hal, Hal-TCT, Hal-TCT-Py, and Hal-Py-SO_3_H, a new band between 1690 and 1699 cm^−1^ is clearly visible, which is associated with TCT and 3-aminopyridine due to their C=N functionality. The FTIR spectra of Hal-TCT and Hal-NH_2_ show an absorbance band at 2927 cm^−1^ associated with -CH_2_, proving conjugation of APTES.

**Figure 6:**
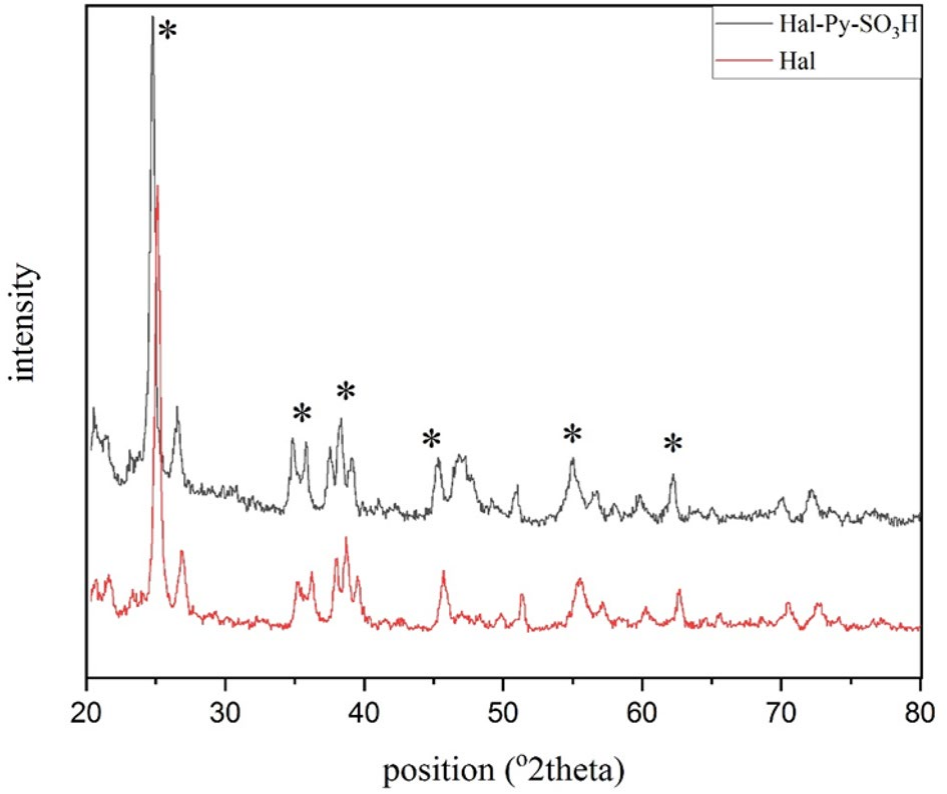
XRD patterns of Hal and Hal-Py-SO_3_H.

Figure 6 shows the XRD analysis of Hal-Py-SO_3_H. According to studies conducted on the XRD spectrum of halloysite, the index peaks occur at 2θ = 8, 12, 25, 35, 56 and 65 (JCPDS card No. 00-029-1487)^28^. In the XRD spectrum of the Hal-Py-SO_3_H catalyst, the peaks associated with halloysite are clearly visible, and its structure is well preserved. The XRD patterns of Hal and Hal-Py-SO_3_H are presented in Fig. 6 for the purpose of conducting a comparison.

**Figure 7:**
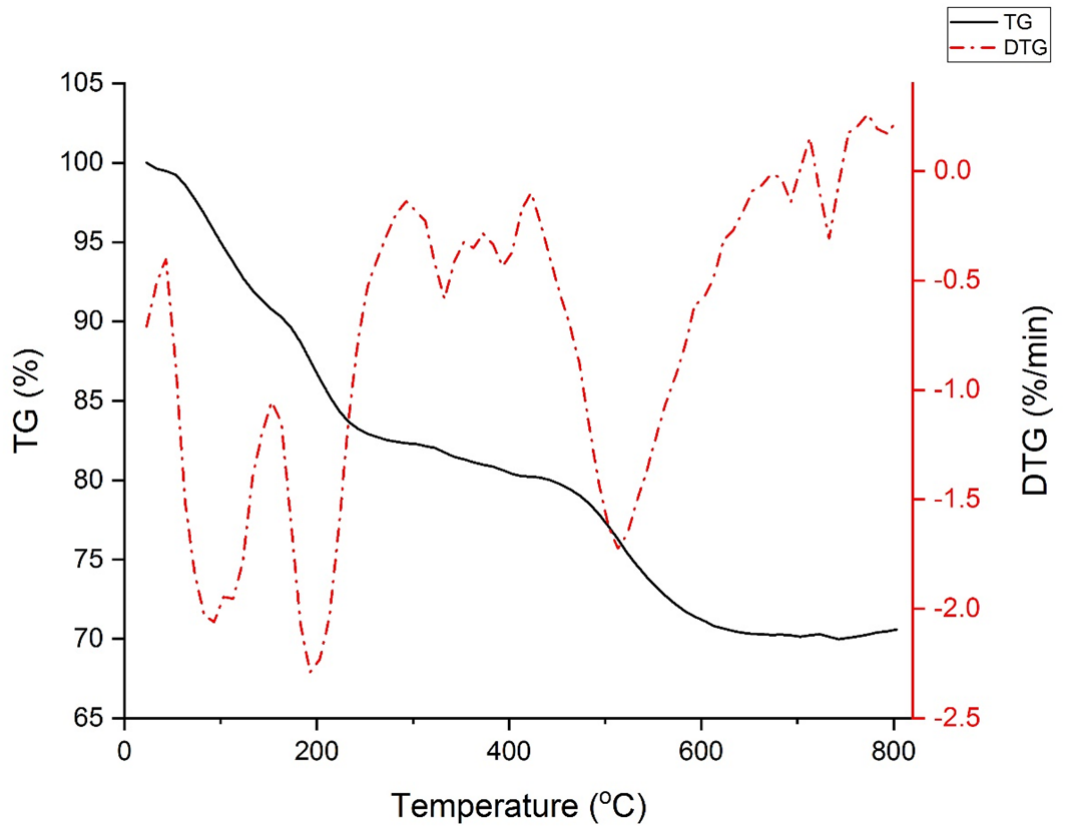
TG thermograms and DTG of Hal-Py-SO_3_H.

In Fig. 7, the TGA diagram of Hal-Py-SO_3_H exhibits an initial weight reduction, which can be attributed to the removal of water absorbed on the surface. At temperatures ranging from 180 to 580 °, a weight loss of 28.19% is observed, which is associated with the loading of the organic ligand on the halloysite material.

### Catalytic activity

The catalytic activity of Hal-Py-SO_3_H was investigated in two different organic reactions. The first one involves reaction of dimedone and benzaldehyde derivatives, and the second involves reaction of ninhydrin, malononitrile, and 1,3-diketones. Initially, the optimal conditions for synthesis of xanthene's derivatives as a two components reaction were determined. Different parameters such as amounts of catalyst, solvent, and temperature were tested to optimize the process. Reaction between dimedone and benzaldehyde was chosen as a model reaction (Table 1). The best reaction condition was optimized as using 0.04 g Hal-Py-SO_3_H in water at reflux for 30 min. (Table 1, entry 5).

**Table 1:** Reaction condition screening for the synthesis of 3,3,6,6-tetramethyl-9-phenyl- 3,4,5,6,7,9- hexahydro-1H-xanthene-1,8(2H)-dione.


Entry	Hal-Py-SO_3_H (mg)	Solvent	Temperature (°C)	Time (min)	Yield %
1	20	H_2_O	Reflux	250	Trace
2	30	H_2_O	Reflux	45	85
3	40	H_2_O	50	150	80
4	40	EtOH	r.t.	270	20
**5**	**40**	**H**_**2**_**O**	**Reflux**	**30**	**96**
6	40	EtOH	Reflux	90	80
7	50	H_2_O	Reflux	35	95
8	40	H_2_O	r.t.	210	30
9	40	H_2_O:EtOH (1:1)	50	185	75
10	40	Acetone	Reflux	120	70
11	40	Acetonitrile	Reflux	220	55
12	40	Toluene	80	270	10

Significant values are in bold.

After obtaining the optimization condition, reaction of different benzaldehyde derivatives and dimedone were investigated (Table 2). 2 mmol of dimedone was mixed with 1 mmol of benzaldehyde derivative, 5 mL of distilled water as a solvent in the presence of 40 mg of catalyst and the reaction progress was monitored by TLC. After the reaction was completed, the precipitate was filtered and dried. Recrystallization in hot ethanol gives the pure product **3a-j**.

**Table 2:** Synthesis of xanthene derivatives.


Entry	Product	Time (min)	Yield %	TON	TOF	m.p. (°C) Obs.	m.p. (°C) Lit
1		30	95	18,445	614	199–201	204–205^29^
2		45	98	19,028	422	210–211	226–228^29^
3		50	95	18,445	368	227–232	231**–**233^29^
4		100	96	18,639	186	226–228	239–241^29^
5		30	98	19,028	634	203–205	205–207^30^
6		30	96	18,639	621	219–220	222–224^29^
7		50	95	18,445	368	239–242	247–248^29^
8		55	97	18,833	342	> 300	320^31^
9		80	98	19,028	237	160–163	168–170^32^
10		40	96	18,639	465	228–229	227–229^33^

In the next step, the catalytic efficiency of the catalyst was assessed in the process of spiropyran synthesis. A summary of the results for investigation of the optimal amount of catalyst, solvent and temperature for the spiropyran synthesis is provided in Table 3.The results indicate that the ideal conditions involved utilizing 0.04 g of Hal-Py-SO_3_H in water as a solvent at reflux temperature. (Table 3, entry 6).

**Table 3:** Reaction condition screening for the synthesis of 2-amino-7,7-dimethyl-1ʹ,3ʹ,5-trioxo- 1ʹ,2,3,3ʹ,5,6,7,8 octahydrospiro [chromene-4,2ʹ-indene]-3-carbonitrile.


Entry	Hal-Py-SO_3_H (mg)	Solvent	Temperature (°C)	Time (min)	Yield%
1	–	H_2_O	Reflux	130	10
2	15	H_2_O	Reflux	110	25
3	25	H_2_O	Reflux	110	30
4	35	H_2_O	Reflux	45	60
5	35	EtOH	r.t.	1440	Trace
**6**	**40**	**H**_**2**_**O**	**Reflux**	**15**	**98**
7	40	EtOH	Reflux	20	95
8	40	H_2_O	r.t.	50	45

Significant values are in bold.

After obtaining the optimization condition, reaction of different 1,3-dikeone derivatives and ninhydrin and malononitrile/ethyl cyanoacetate were investigated (Table 4). 1 mmol of ninhydrin was mixed with 1 mmol of malononitrile/ethyl cyanoacetate and 1 mmol various of 1,3-diketone compound, 5 mL of distilled water as a solvent in the presence of 40 mg of catalyst and the reaction progress was monitored by TLC. Recrystallization in hot ethanol gives the pure product.

**Table 4:** Synthesis of spiropyran derivatives.


Entry	Product	R	Time (min)	Yield %	TON	TOF	m.p. (°C) Obs.	m.p. (°C) Lit
1		CN	15	98	19,027	1268	290–292	295–297^36^
2		CN	20	95	18,445	922	277–280	278–280^12^
3		CN	15	97	18,833	1255	250–255	280^36^
4		CN	15	96	18,639	1242	282–287	290–295^34^
5		CN	40	94	18,251	456	249–252	251–253^12^
6		CN	60	93	18,057	300	273–276	275–277^16^
7		CO_2_Et	10	98	19,027	1902	207–211	215^34^
8		CO_2_Et	80	92	17,862	223	224–227	225–227^16^

Tables 2 and 4 contain the recorded results of the calculations performed for TON and TOF. The molar mass of halloysite poses a challenge in terms of accurate calculation as a result of the existence of water groups. Nonetheless, an approximation for the molar mass of the catalyst has been determined.

**Figure 8:**
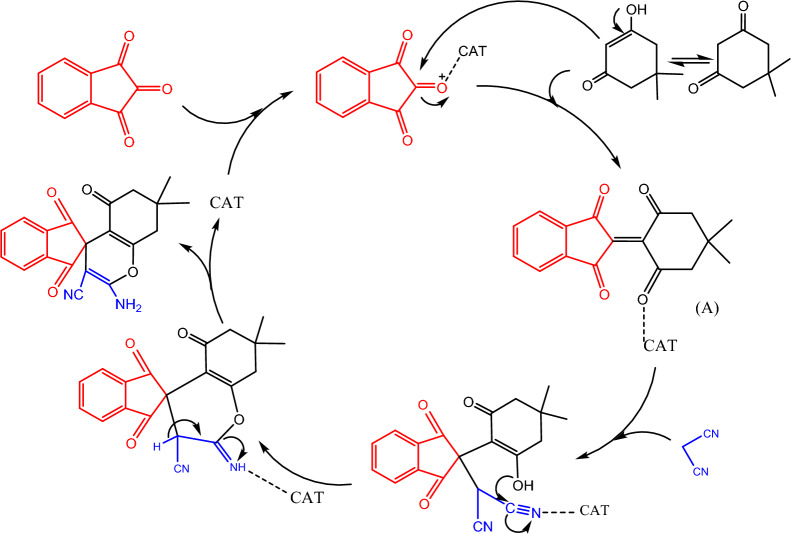
Suggested probable mechanism for the synthesis of spiropyrans.

As seen in Fig. 8, a proposed mechanism involves an aldol condensation between ninhydrin and 1,3-dicarbonyl compounds, resulting in an intermediate A. Michael addition of molononitrile to intermediate A, following by an intramolecular cyclization, gives the final product. Hal-Py-SO_3_H is capable of activating the aldehyde in order to increase the aldol condensation.

### Hot filtration test

Two avenues exist for heterogeneous catalysis. In the first scenario, known as true heterogeneous catalysis, the catalytic species is expected to be immobilized on the support, whereas in the alternative scenario, the catalytic species may leach into the reaction medium. A widely recognized method for discerning the catalyst's nature is hot filtration, where the catalyst is removed from the reaction after a short period, and then the reaction proceeds without it. In true heterogeneous catalysis, where no leaching occurs, the reaction does not proceed after catalyst removal. Conversely, in the latter case, the reaction progresses in the absence of the catalyst due to leached species. To validate the efficacy of the Hal-Py-SO_3_H catalyst, a hot filtration test was conducted in a model reaction (2 mmol dimedone and 1 mmol benzaldehyde), confirming its status as a true heterogeneous catalyst. Specifically, the corresponding xanthene product was not produced after the catalyst was removed from the reaction medium.

### Comparative study

The activity of Hal-Py-SO_3_H catalyst was compared with other reported catalysts for manufacturing spiropyrans in Table 5. As shown, by comparing of Hal-Py-SO_3_H with other previous catalysts, it can be considered comparable in terms of its catalytic performance.

**Table 5:** Comparison of Hal-Py-SO_3_H activity with other catalysts for the synthesis of spiropyrans.

Entry	Catalyst	Catalyst amount	Solvent	Temperature (°C)	Time (min)	Yield (%)
1	NiFe_2_O_4_@SiO_2_@Melamine	0.025 g	EtOH	Reflux	15	95^12^
2	Poly(Ani-co-Py)@CNT-Fe_3_O_4_	0.04 g	H_2_O	80	120	85^35^
3	PS@GO-Fe_3_O_4_	0.01 g	H_2_O	80	120	93^36^
4	Na_2_EDTA	15 mol%	–	70	12	92^37^
5	NaHCO_3_	0.02 mmol	EtOH	Reflux	20	92^38^
6	Hal-Py-SO_3_H	0.04 g	H_2_O	Reflux	15	98

### Reusability of catalyst

**Figure 9:**
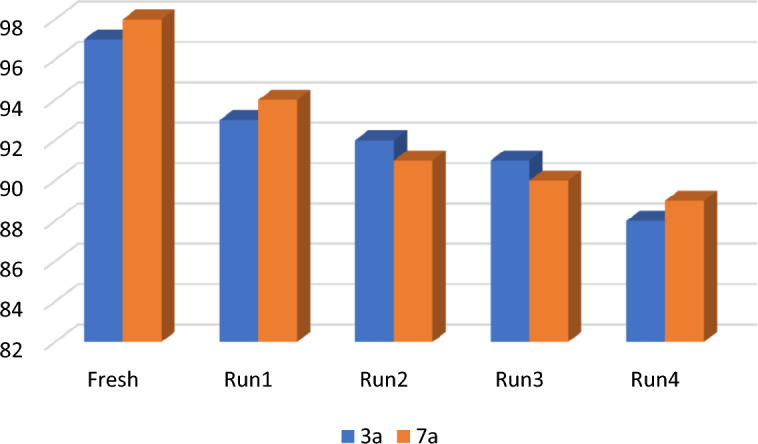
The examination of the reusability of Hal-Py-SO_3_H.

The Hal-Py-SO_3_H evaluated for its recyclability and reusability. To achieve this goal, reaction of ninhydrin, dimedone, and malononitrile was selected as a model reaction for the synthesis of (**3a**) under the optimal reaction conditions. Moreover, synthesis of (**7a**) has been also regarded as a model reaction for spiropyran compounds. The recovered catalyst was reused in the model reaction under similar reaction conditions in the following run. As seen in Fig. 9, the catalyst could be recovered and reused at five runs without any significant loss of catalytic performance.

## Conclusion

In conclusion, we have presented the development of Hal-Py-SO_3_H as an innovative sulfonic acid catalyst. The synthesis of various spiropyran derivatives has been effectively achieved through a facile three-component one-pot reaction employing ninhydrin, malononitrile / ethyl cyanoacetate, and 1,3-diketone compounds. This process has been carried out under reflux conditions in the presence of a reusable catalyst, offering notable advantages such as environmentally-friendly characteristics, gentle reaction conditions, and short reaction durations. Importantly, the resulting products exhibit significant potential in the fields of pharmaceutical and biological sciences. Furthermore, it is worth noting that the catalytic activity of the nano catalysts remains largely unaffected even after multiple reuses, with only negligible traces of activity lost.

### Supplementary Information

Supplementary Information.

## Data Availability

All data generated or analysed during this study are included in this published article and its supplementary information file.

## References

[1] Chen TL, Kim H, Pan SY, Tseng PC, Lin YP, Chiang PC (2020). Implementation of green chemistry principles in circular economy system towards sustainable development goals: Challenges and perspectives. Sci. Total Environ..

[2] Daraie M, Heravi MM, Rangraz Y, Besharati Z (2021). Pd NPs supported on halloysite functionalized with Schiff base as an efficient catalyst for Sonogashira reaction. Sci. Rep..

[3] Polshettiwar V, Varma RS (2010). Green chemistry by nano-catalysis. Green Chem..

[4] Anastas PT, Bartlett LB, Kirchhoff MM, Williamson TC (2000). The role of catalysis in the design, development, and implementation of green chemistry Paul. Catal. Today.

[5] Moeinpour F, Dorostkar-Ahmadi N, Sardashti-Birjandi A, Khojastehnezhad A, Vafaei M (2014). Multicomponent preparation of 1-amidoalkyl-2-naphthols using silica-supported molybdenum oxide (MoO_3_/SiO_2_) as a mild and recyclable catalyst. Res. Chem. Intermed..

[6] Zhang Y, Ouyang J, Yang H (2014). Metal oxide nanoparticles deposited onto carbon-coated halloysite nanotubes. Appl. Clay Sci..

[7] Zhang Y, He X, Ouyang J, Yang H (2013). Palladium nanoparticles deposited on silanized halloysite nanotubes: Synthesis, characterization and enhanced catalytic property. Sci. Rep..

[8] Sadjadi S, Heravi MM, Daraie M (2017). Heteropolyacid supported on amine-functionalized halloysite nano clay as an efficient catalyst for the synthesis of pyrazolopyranopyrimidines via four-component domino reaction. Res. Chem. Intermed..

[9] Yuan P, Southon P, Liu Z, Green M, Hook J, Antill S, Kepert C (2008). Functionalization of halloysite clay nanotubes by grafting with γ-aminopropyltriethoxysilane. J. Phys. Chem. C.

[10] Cao XT, Showkat AM, Kim DW, Jeong YT, Kim JS, Lim KT (2015). Preparation of β-cyclodextrin multi-decorated halloysite nanotubes as a catalyst and nanoadsorbent for dye removal. J. Nanosci. Nanotechnol..

[11] Shi B, Li Y, Zhang H, Wu W, Ding R, Dang J, Wang J (2016). Tuning the performance of anion exchange membranes by embedding multifunctional nanotubes into a polymer matrix. J. Membr. Sci..

[12] Hosseini Nasab N, Safari J (2019). Synthesis of a wide range of biologically important spiropyrans and spiroacenaphthylenes, using NiFe_2_O_4_@SiO_2_@Melamine magnetic nanoparticles as an efficient, green and reusable nanocatalyst. J. Mol. Struct..

[13] Mohamadpour F, Maghsoodlou MT, Heydari R, Lashkari M (2016). Copper(II) acetate monohydrate: An efficient and eco-friendly catalyst for the one-pot multi-component synthesis of biologically active spiropyrans and 1H-pyrazolo[1,2-b]phthalazine-5,10-dione derivatives under solvent-free conditions. Res. Chem. Intermed..

[14] Safari F, Hosseini H, Bayat M, Ranjbar A (2019). Synthesis and evaluation of antimicrobial activity, cytotoxic and pro-apoptotic effects of novel spiro-4 H-pyran derivatives. RSC Adv..

[15] Ahadi N, Bodaghifard MA, Mobinikhaledi A (2019). Cu (II)-β-cyclodextrin complex stabilized on magnetic nanoparticles: A retrievable hybrid promoter for green synthesis of spiropyrans. Appl. Organomet. Chem..

[16] Babar K, Zahoor AF, Ahmad S, Akhtar R (2021). Recent synthetic strategies toward the synthesis of spirocyclic compounds comprising six-membered carbocyclic/heterocyclic ring systems. Mol. Divers..

[17] Mosslemin MH, Anary-Abbasinejad M, Anaraki-Ardakani H (2009). Reaction between isocyanides, dialkyl acetylenedicarboxylates and 2-hydroxy-1-aryl-2-(arylamino)ethanones: One-pot synthesis of highly functionalized 2-aminofurans. Synlett..

[18] Insuasty D, Castillo J, Becerra D, Rojas H, Abonia R (2020). Synthesis of biologically active molecules through multicomponent reactions. Molecules.

[19] Das S (2020). Recent applications of ninhydrin in multicomponent reactions. RSC Adv..

[20] Khandelwal, S., Tailor, Y. K., Rushell, E. & Kumar, M. Use of sustainable organic transformations in the construction of heterocyclic scaffolds. In *Green Approaches in Medicinal Chemistry for Sustainable Drug Design* 245–352 (Elsevier, 2020) 10.1016/B978-0-12-817592-7.00009-5.

[21] Allameh S, Davoodnia A, Khojastehnezhad A (2012). An efficient and eco-friendly synthesis of 14-aryl-14H-dibenzo[a, j]xanthenes using _H_4[Si_W1_2_O4_0] as a heterogeneous and reusable catalyst under solvent-free conditions. Chin. Chem. Lett..

[22] Appaturi JN, Ratti R, Phoon BL, Batagarawa SM, Din IU, Selvaraj M, Ramalingam RJ (2021). A review of the recent progress on heterogeneous catalysts for Knoevenagel condensation. Dalton Trans..

[23] Lin L, Han X, Han B, Yang S (2021). Emerging heterogeneous catalysts for biomass conversion: Studies of the reaction mechanism. Chem. Soc. Rev..

[24] Zhang S, Gao H, Huang Y, Wang X, Hayat T, Li J, Xu X, Wang X (2018). Ultrathin g-C_3_N_4_ nanosheets coupled with amorphous Cu-doped FeOOH nanoclusters as 2D/0D heterogeneous catalysts for water remediation. Environ. Sci. Nano.

[25] Zhang Y, Yang G-W, Xie R, Yang L, Li B, Wu G-P (2020). Scalable, durable, and recyclable metal-free catalysts for highly efficient conversion of CO_2_ to cyclic carbonates. Angew. Chemie.

[26] Sadjadi S, Koohestani F, Abedian-Dehaghani N, Heravi MM (2021). Halloysite nanoclay with high content of sulfonic acid-based ionic liquid: A novel catalyst for the synthesis of tetrahydrobenzo[b]pyrans. Catalysts.

[27] Massaro M, Colletti CG, Lazzara G, Milioto S, Noto R, Riela S (2017). Halloysite nanotubes as support for metal-based catalysts. J. Mater. Chem. A.

[28] Maleki A, Hajizadeh Z, Firouzi-Haji R (2018). Eco-friendly functionalization of magnetic halloysite nanotube with SO_3_H for synthesis of dihydropyrimidinones. Microporous Mesoporous Mater..

[29] Zolfagharinia S, Koukabi N, Kolvari E (2016). A unique opportunity for the utilization of glass wastes as a resource for catalytic applications: Toward a cleaner environment. RSC Adv..

[30] Shashi R, Begum NS, Panday AK (2021). A rapid ultrasound synthesis of xanthenediones catalyzed by boric acid in ethanol-water medium: Single crystal, DFT and Hirshfeld surface analysis of two representative compounds. J. Mol. Struct..

[31] Cremlyn RJ, Saunders D (1993). Chlorosulfonation of 9-aryl 3,3,6,6-Tetramethyloctahydroxanthen-1,8-diones. Phosphorus. Sulfur. Silicon Relat. Elem..

[32] Shaikh SA, Kamble VS, Salunkhe ST, Patil SK, Aghav BD (2023). Efficient synthesis of xanthenediones using CuCeO_2_ nanoparticle catalyst in aqueous medium. Org. Prep. Proced. Int..

[33] Zukić S, Veljović E, Špirtović-Halilović S, Muratović S, Osmanović A, Trifunović S, Novaković I, Završnik D (2018). Antioxidant, antimicrobial and antiproliferative activities of synthesized 2,2,5,5-tetramethyl-9-aryl-3,4,5,6,7,9-hexahydro-1H-xanthene-1,8(2H)-dione derivatives. Croat. Chem. Acta.

[34] Darehkordi A, Karimi-Taleghani Z, Pouralimardan OA (2014). One-pot of three-component synthesis of novel amino-spiroindene derivatives. J. Iran. Chem. Soc..

[35] Hojati SF, Amiri A, Fardi E, Mahamed M (2022). The copolymer coating effect on the catalytic activity of magnetic carbon nanotube (CNT-Fe_3_O_4_) in the multi-component reactions. Res. Chem. Intermed..

[36] Hojati SF, Amiri A, Mahamed M (2020). Polystyrene@graphene oxide-Fe_3_O_4_ as a novel and magnetically recyclable nanocatalyst for the efficient multi-component synthesis of spiro indene derivatives Seyedeh. Res. Chem..

[37] Jazinizadeh T, Maghsoodlou MT, Heydari R, Yazdani-Elah-Abadi A (2017). Na_2_EDTA: An efficient, green and reusable catalyst for the synthesis of biologically important spirooxindoles, spiroacenaphthylenes and spiro-2-amino-4H-pyrans under solvent-free conditions. J. Iran. Chem. Soc..

[38] He Y, Guo H, Tian J (2011). A simple three-component synthesis of spiro-pyran derivatives. J. Chem. Res..

