# Supplementary material for: Hal-Py-SO3H as a novel and recyclable catalyst for highly efficient synthesis of xanthene and spiropyran derivatives
Source: Sci Rep. 2024 Apr 6;14:8085. doi: 10.1038/s41598-024-58647-x (PMC10998835; doi:10.1038/s41598-024-58647-x)
Supplement: Supplementary file 1 — Supplementary Information. [file 41598_2024_58647_MOESM1_ESM.pdf]

Hal-Py-SO_3H as a novel and recyclable catalyst for highly efficient synthesis of xanthene and spiropyran derivatives

Abdolmaleki, Mohammad; Daraie, Mansoureh;* Mirjafary, Zohreh*

[a]Department of Chemistry, Science and Research Branch, Islamic Azad University, Tehran, Iran

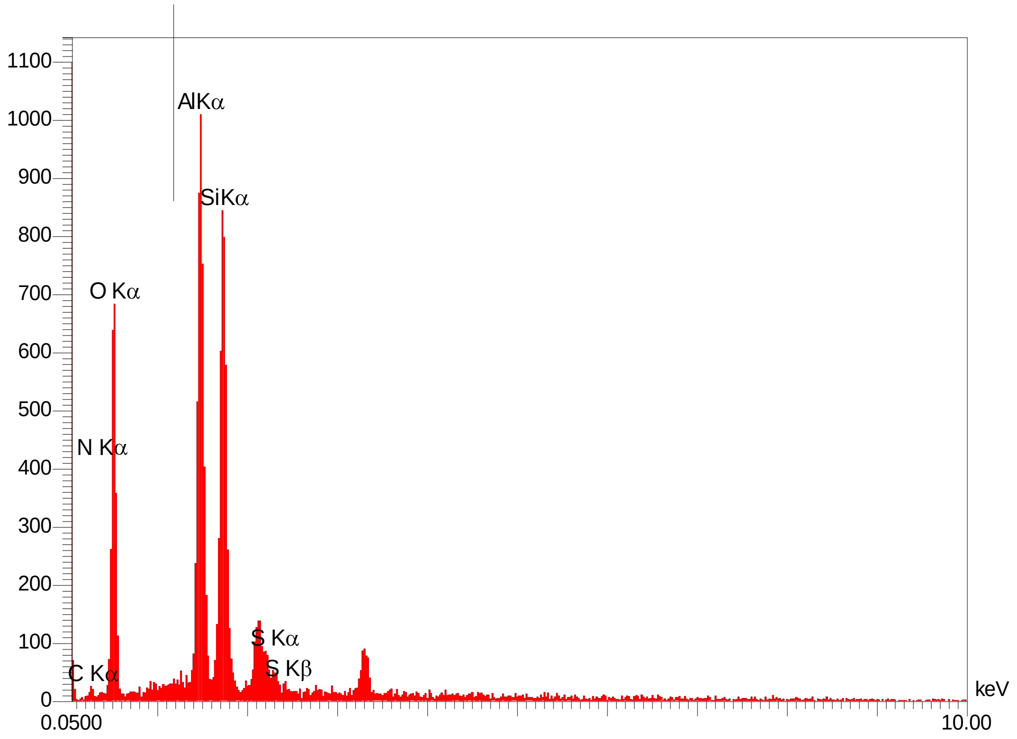

Figure 1. EDS analysis of Hal-Py-SO_3H

Table 1. Quantitative Results of EDS

Elt	Line	Int	Error	K	Kr	W%	A%	ZAF	Ox %	Pk/Bg	Class	LConf	HConf	Cat#
**C**	Ka	1.9	1.4254	0.0254	0.0120	8.17	12.33	0.1466	0.00	12.04	A	6.00	10.33	0.00
**N**	Ka	1.4	1.4490	0.0255	0.0120	5.02	6.50	0.2397	0.00	7.08	A	3.48	6.56	0.00
**O**	Ka	59.3	1.4726	0.3950	0.1863	50.83	57.62	0.3666	0.00	325.46	A	48.42	53.24	0.00
**Al**	Ka	115.2	1.4822	0.2691	0.1269	17.17	11.54	0.7395	0.00	31.09	A	16.58	17.75	0.00
**Si**	Ka	105.2	1.5041	0.2569	0.1212	17.07	11.02	0.7101	0.00	37.78	A	16.46	17.67	0.00
**S**	Ka	9.0	27.6226	0.0281	0.0132	1.75	0.99	0.7554	0.00	5.68	A	1.54	1.97	0.00
				1.0000	0.4717	100.00	100.00		0.00					0.00

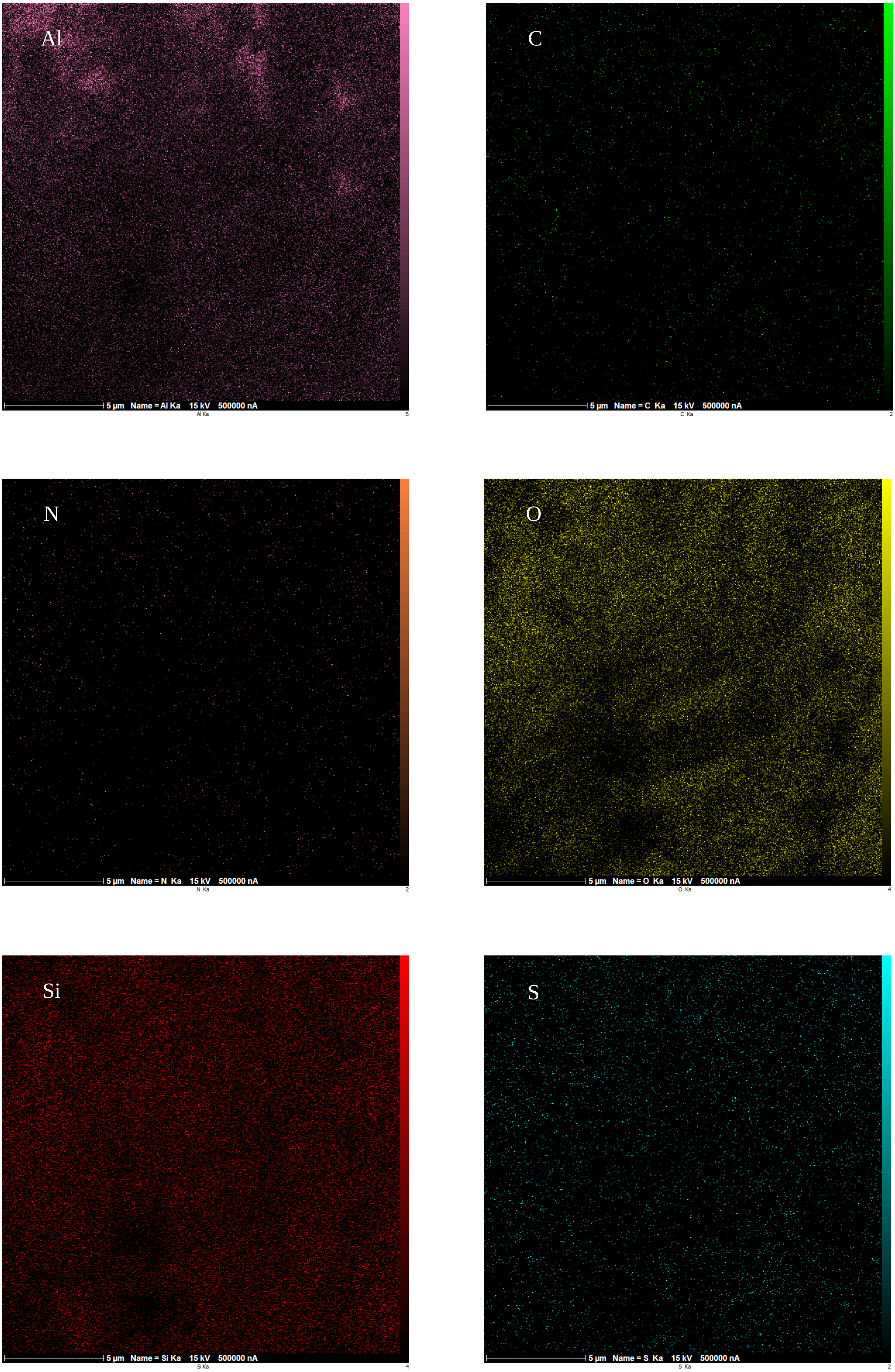

Figure 2. elemental mapping analysis of Hal-Py-SO_3H

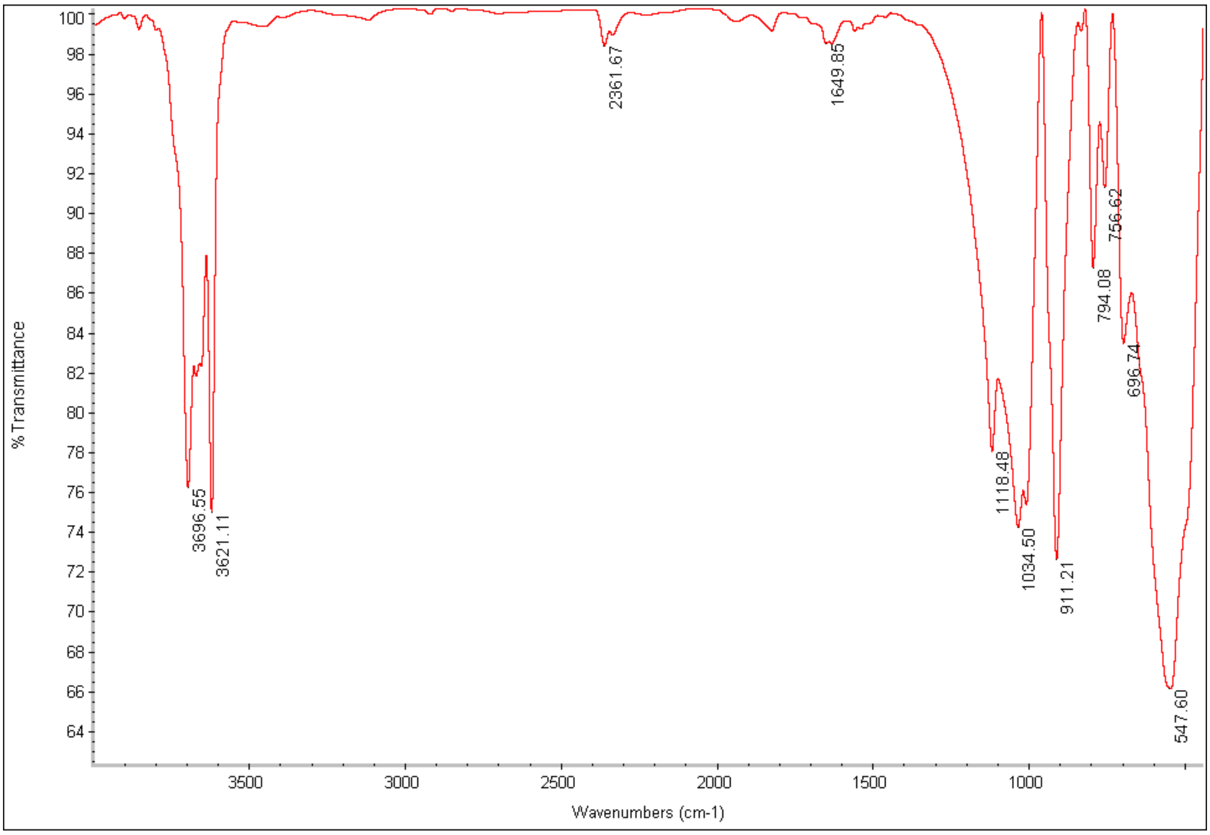

Figure 3. FTIR spectra of Halloysite

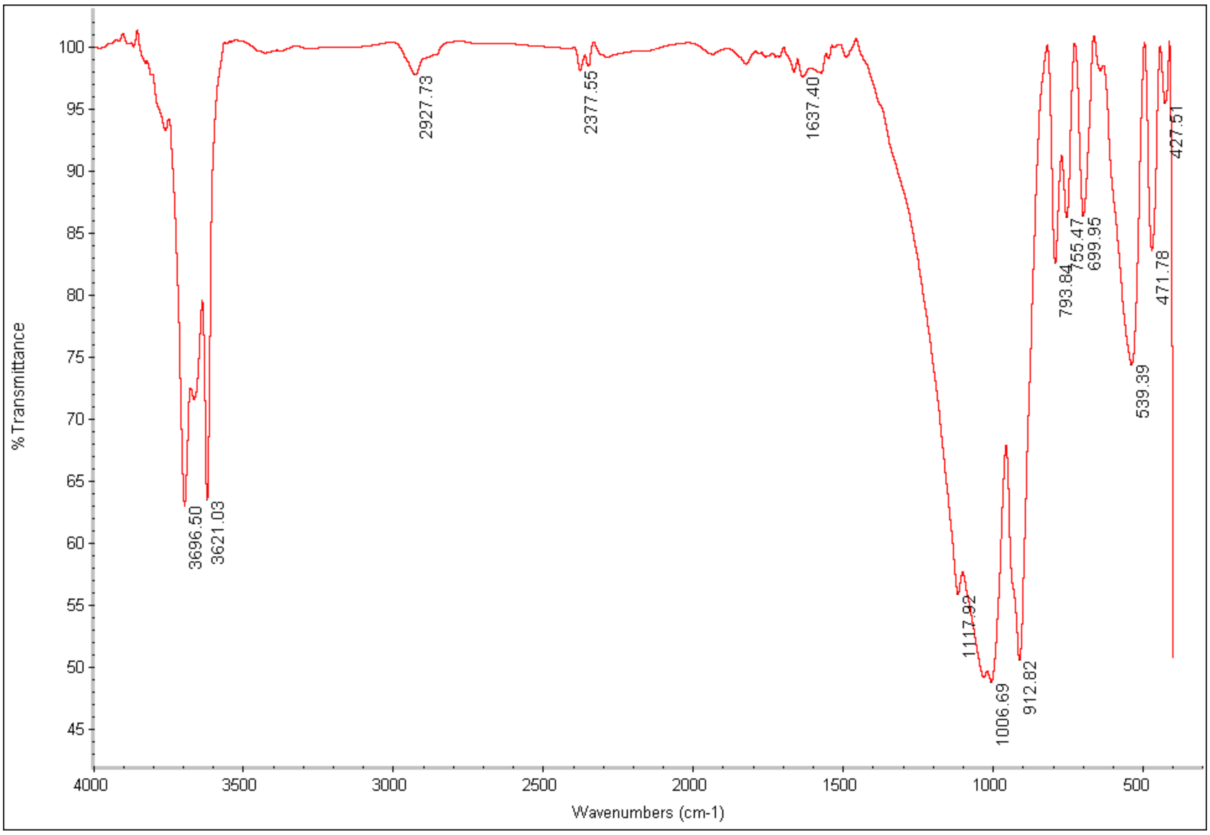

Figure 4. FTIR spectra of Hal-NH_2

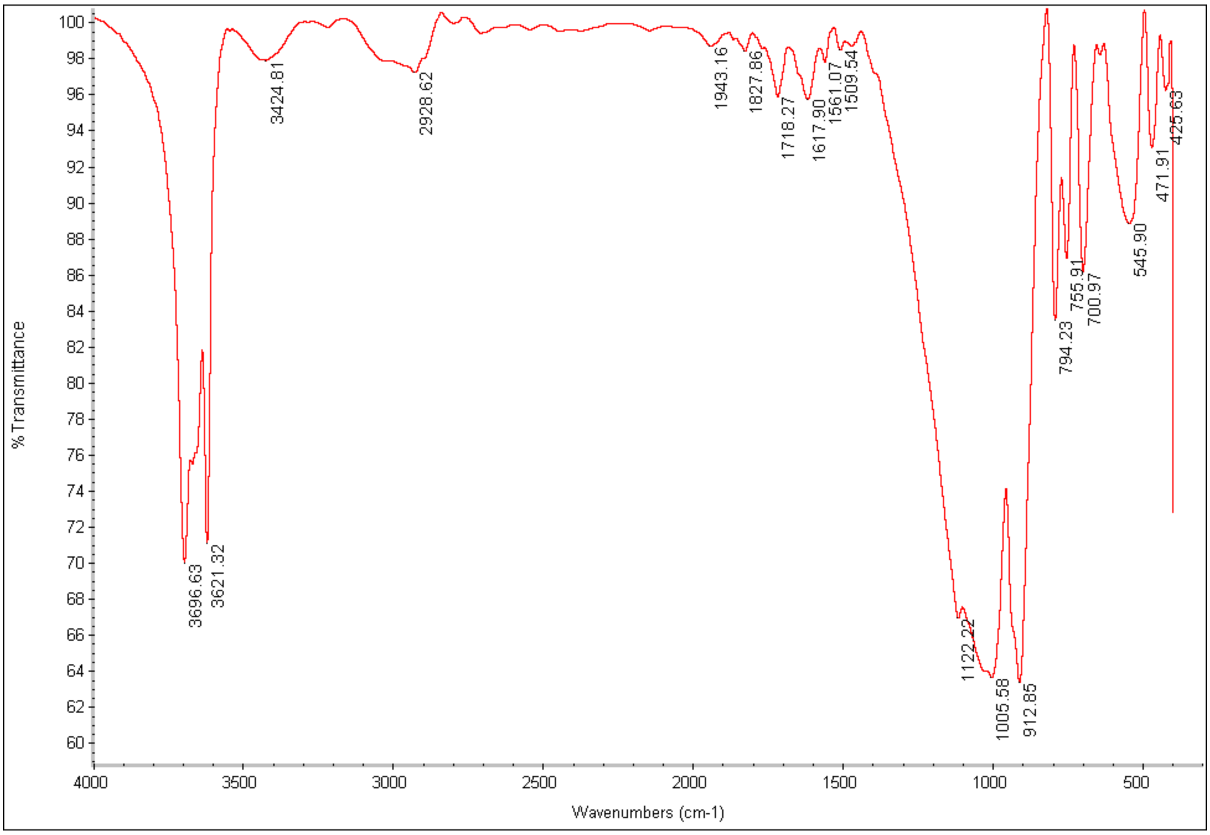

Figure 5. FTIR spectra of Hal-TCT

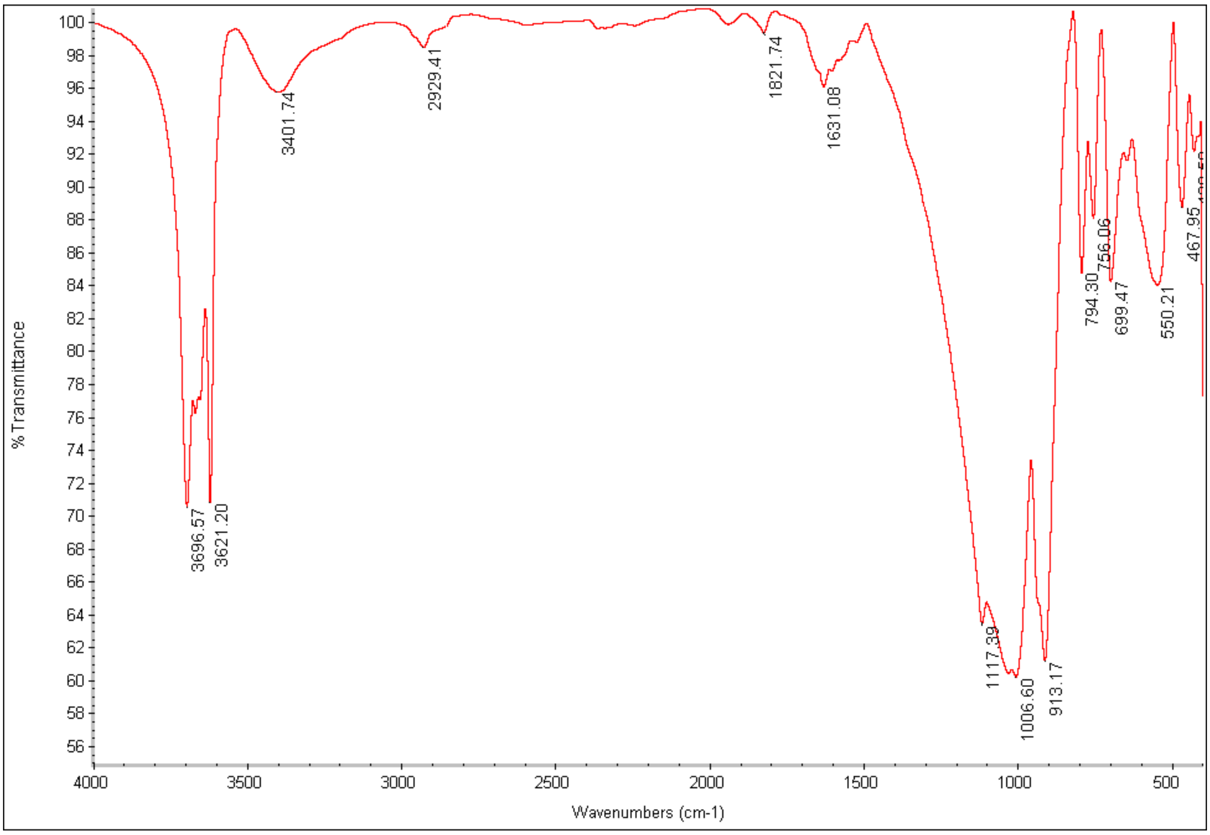

Figure 6. FTIR spectra of Hal-TCT-Py

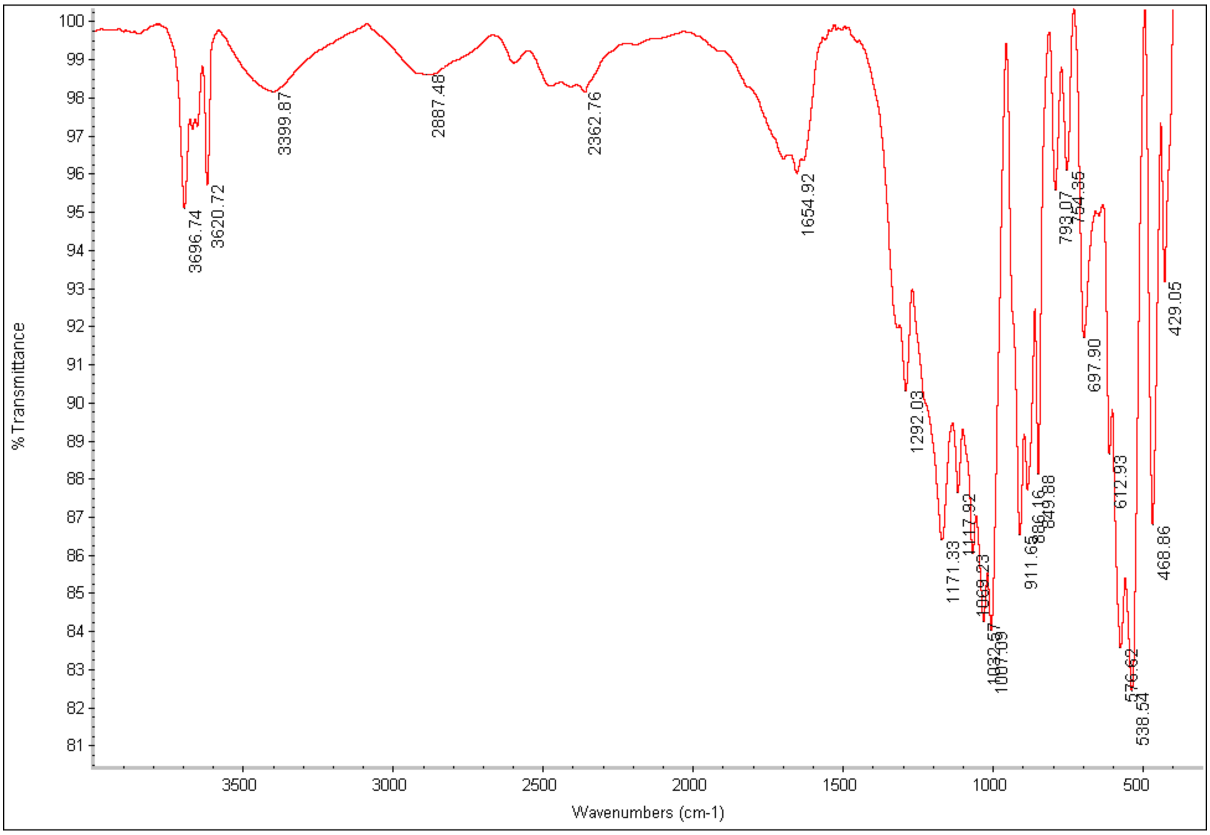

Figure 7. FTIR spectra of Hal-Py-SO_3H

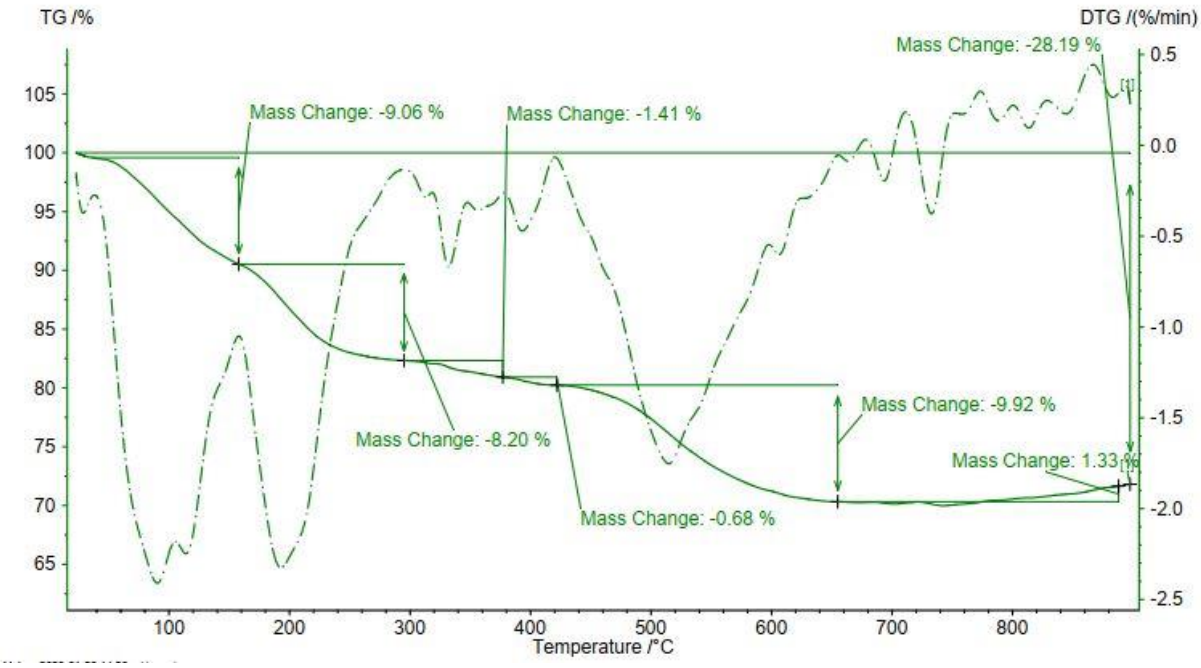

Figure 8. TG thermograms of Hal-Py-SO_3H.

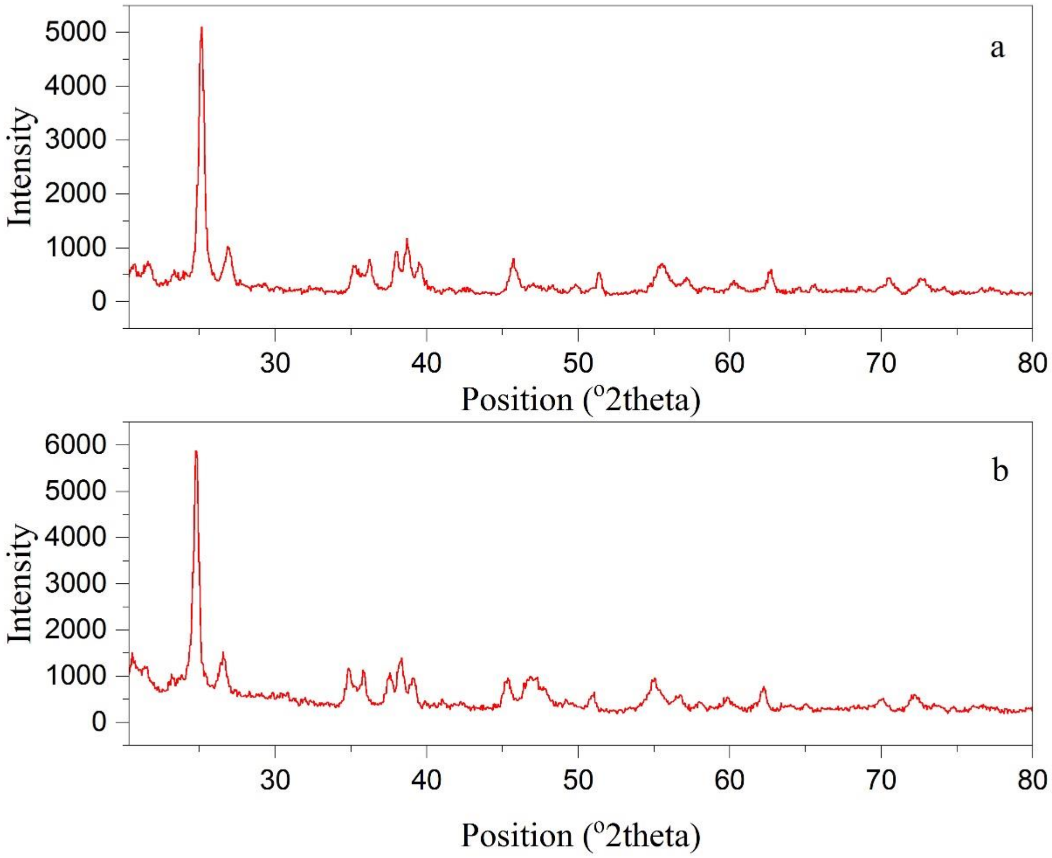

Figure 9. XRD patterns of (a) Hal and (b) Hal-Py-SO_3H.

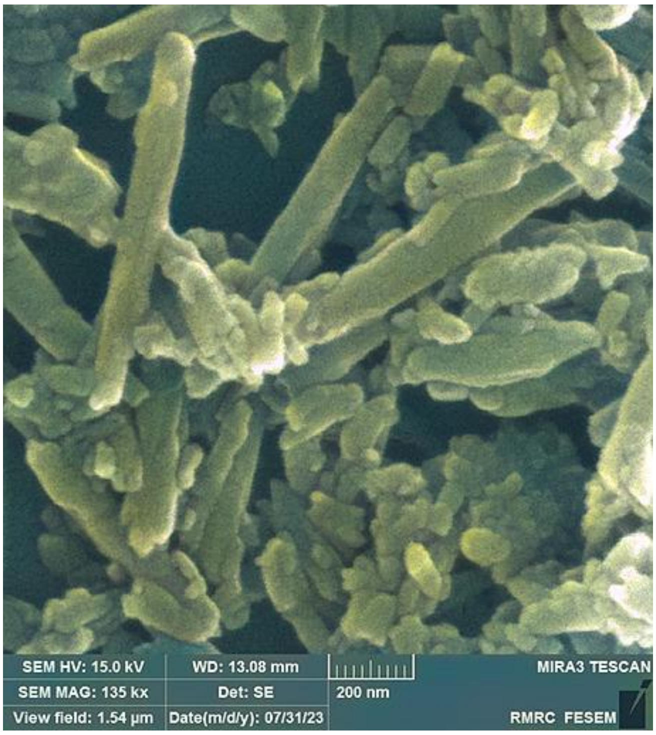

Figure 10. SEM image of Hal-Py-SO_3H

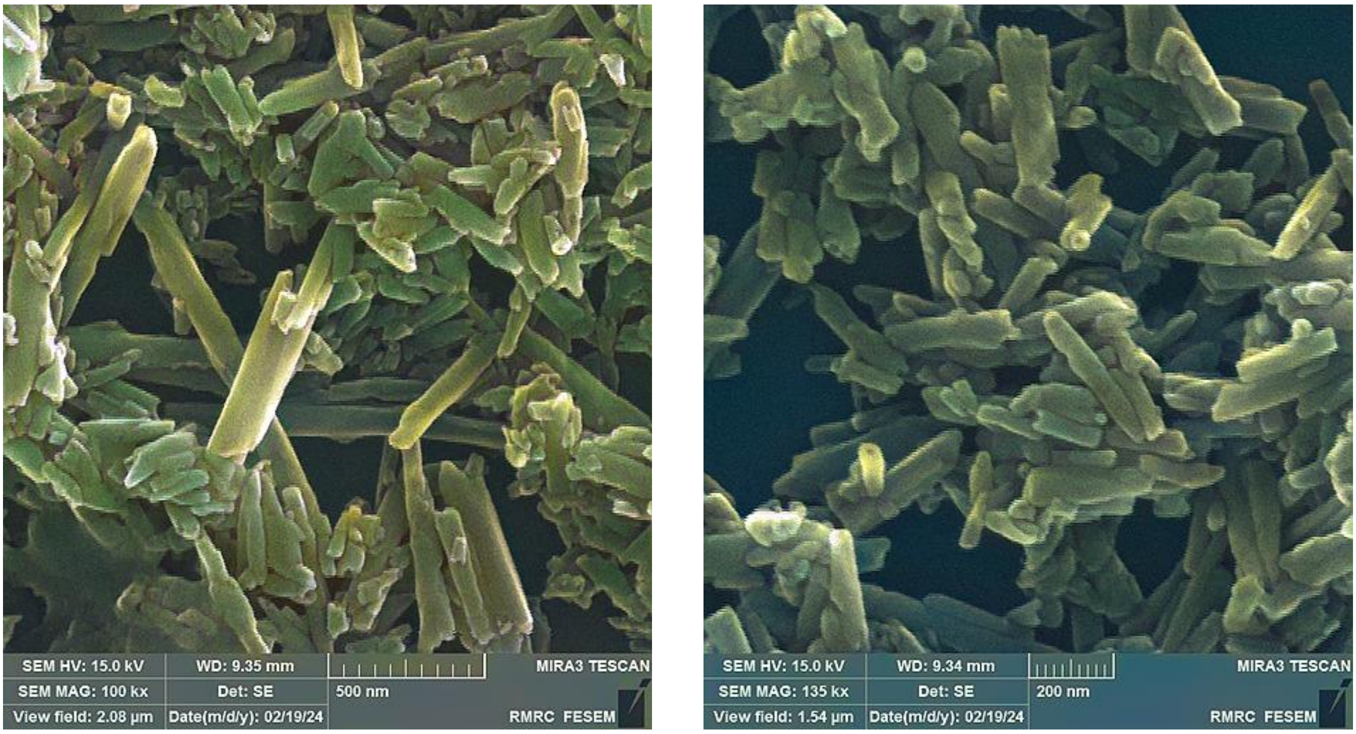

Figure 11. SEM image of Hal

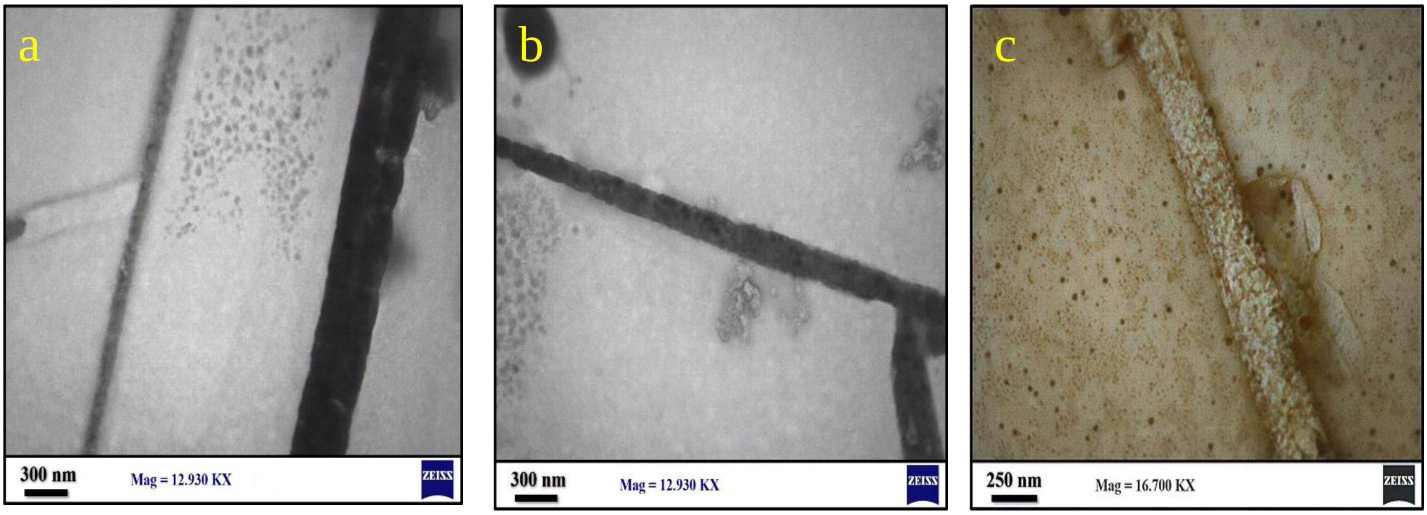

Figure 12. TEM images of (a) Halloysite and (b,c) Hal-Py-SO_3H

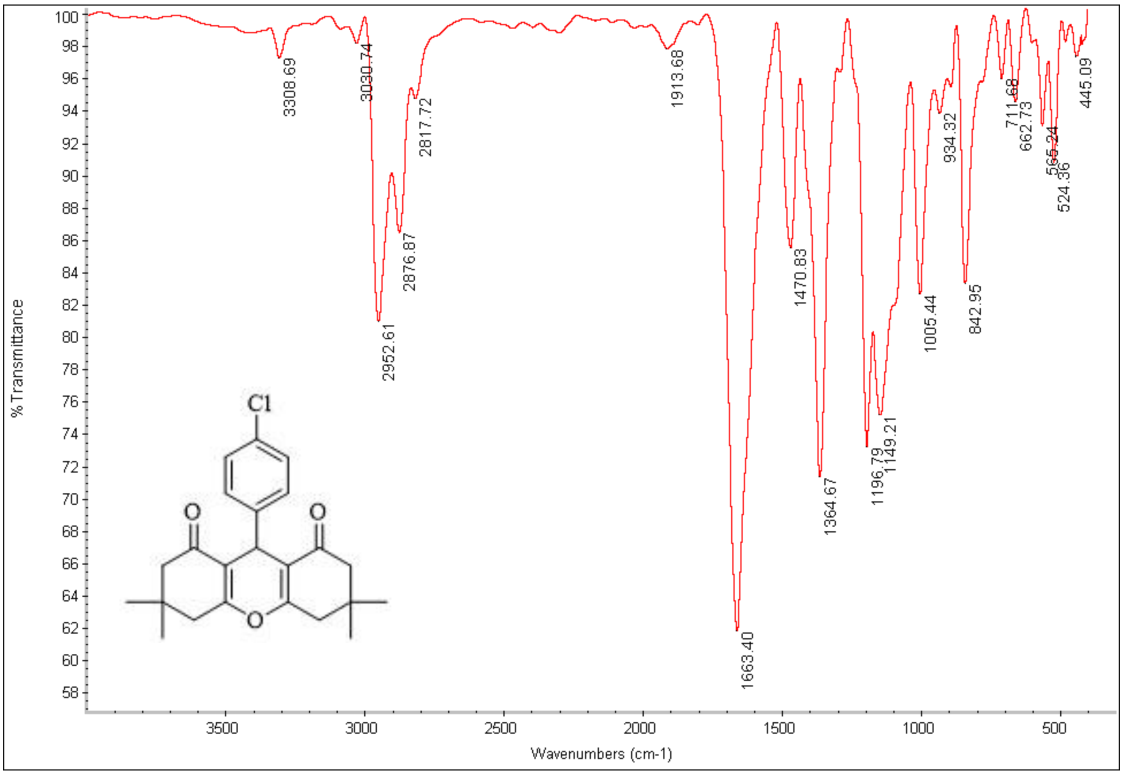

Figure 13. FTIR spectra of 9-(4-chlorophenyl)-3,3,6,6-tetramethyl-3,4,5,6,7,9-hexahydro-1H-xanthene-1,8(2H)-dione (**3c**)

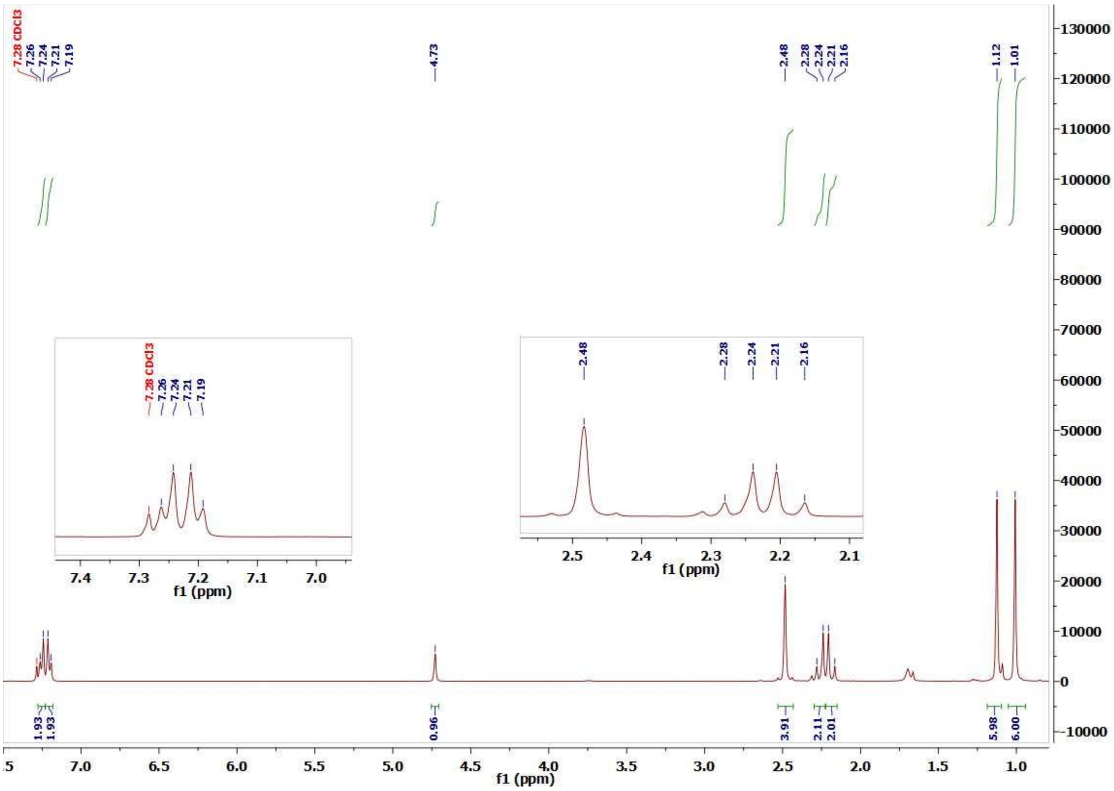

Figure 14. ^{1}HNMR spectra of 9-(4-chlorophenyl)-3,3,6,6-tetramethyl-3,4,5,6,7,9-hexahydro-1H-xanthene-1,8(2H)-dione (**3c**)

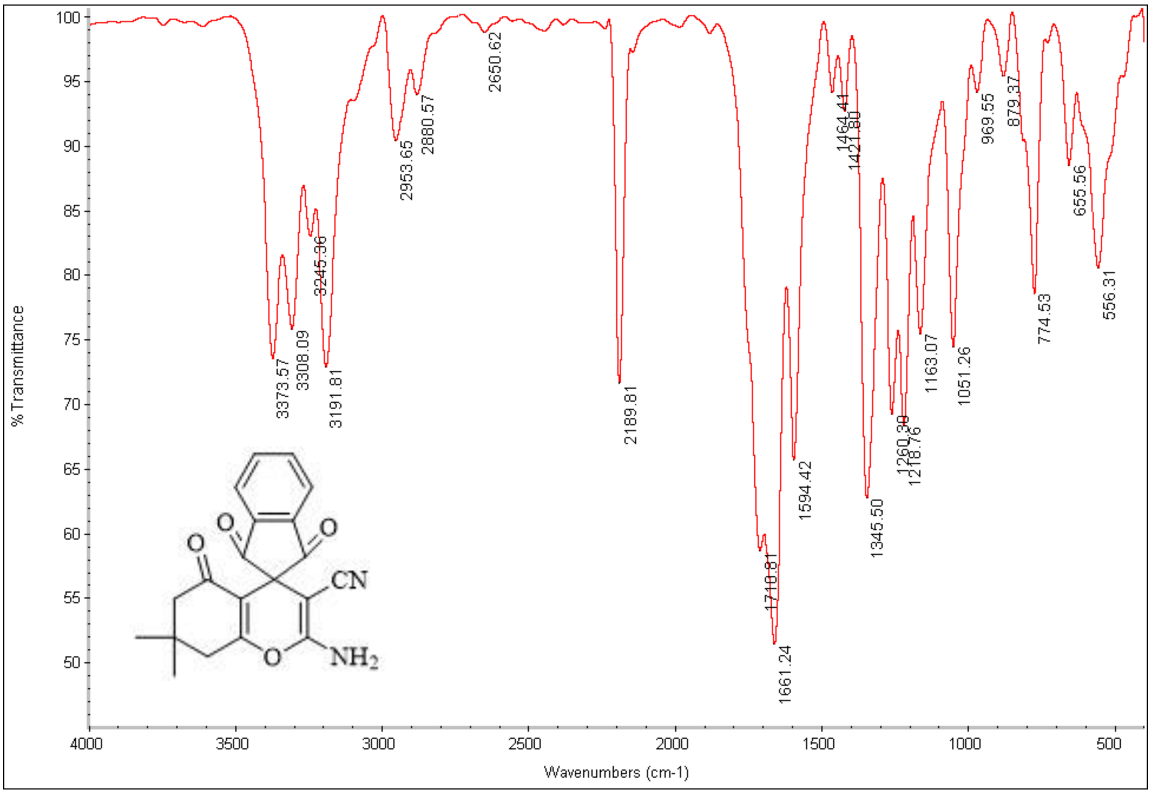

Figure 15. FTIR spectra of 2-amino-7,7-dimethyl-1',3',5-trioxo-1',3',5,6,7,8-hexahydrospiro[chromene-4,2'-indene]-3-carbonitrile (**7a**)

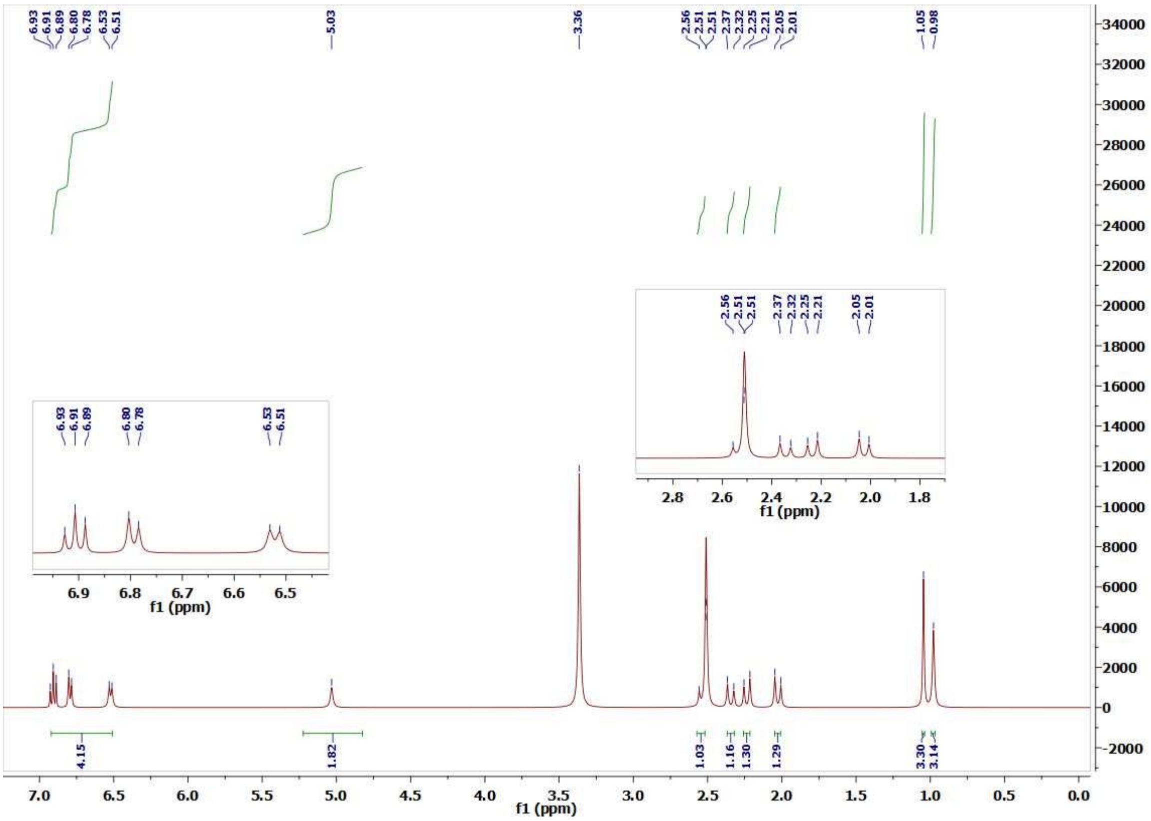

Figure 16. ^{1}HNMR spectra of 2-amino-7,7-dimethyl-1',3',5-trioxo-1',3',5,6,7,8-hexahydrospiro[chromene-4,2'-indene]-3-carbonitrile (**7a**)
